# (1*S*,2*S*,4*R*)-3,3-Dichloro-4,8,12,12-tetra­methyl­tricyclo­[5.5.0.0^2,4^]dodeca-6,8-diene

**DOI:** 10.1107/S1600536810034070

**Published:** 2010-08-28

**Authors:** Ahmed Benharref, Lahcen El Ammari, Moha Berraho, Esaadia Lassaba

**Affiliations:** a Laboratoire de Chimie des Substances Naturelles, "Unité Associé au CNRST (URAC16)", Faculté des Sciences Semlalia, BP 2390 Bd My Abdellah, 40000 Marrakech, Morocco; b Laboratoire de Chimie du Solide Appliquée, Faculté des Sciences, Avenue Ibn Battouta BP 1014 Rabat, Morocco

## Abstract

The title compound, C_16_H_22_Cl_2_, a derivative of β-himachalene, was semi-synthesized from natural essential oils of *Cedrus atlantica*. The mol­ecule is built up from two fused six- and seven-membered rings. The six-membered ring has a perfect chair conformation, whereas the seven-membered ring displays a screw boat conformation; the dihedral angle between the rings is 46.48 (9)°.

## Related literature

For background to himachalene derivatives, see: Plattier & Teiseire (1974 ▶); Sbai *et al.* (2002 ▶). For ring puckering analysis, see: Cremer & Pople (1975 ▶). For the synthesis of the title compound, see: Lassaba *et al.* (1997 ▶). For the reactivity of this sesquiterpene, see: El Jamili *et al.* (2002 ▶; Sbai *et al.* (2002 ▶). For the olfactive properties of β-himachalene, see: Benharref *et al.* (1991 ▶); Bisarya & Dev (1968 ▶); Chekroun *et al.* (2000 ▶).
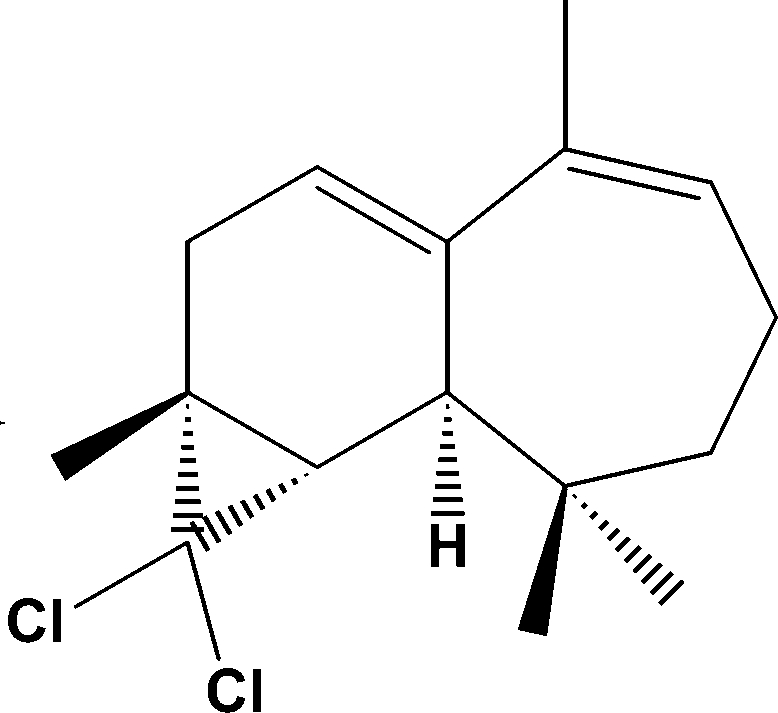


## Experimental

### 

#### Crystal data

C_16_H_22_Cl_2_
*M* *_r_* = 285.24
Orthorhombic, 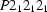

*a* = 7.4356 (17) Å
*b* = 8.3124 (18) Å
*c* = 24.108 (6) Å
*V* = 1490.1 (6) Å^3^
*Z* = 4
Mo *K*α radiation
μ = 0.42 mm^−1^
*T* = 298 K
0.27 × 0.18 × 0.12 mm

#### Data collection

Bruker X8 APEXII CCD area-detector diffractometer
10992 measured reflections
3691 independent reflections
3282 reflections with *I* > 2σ(*I*)
*R*_int_ = 0.026

#### Refinement

*R*[*F* ^2^ > 2σ(*F* ^2^)] = 0.032
*wR*(*F* ^2^) = 0.096
*S* = 1.11
3691 reflections
175 parameters
H atoms treated by a mixture of independent and constrained refinement
Δρ_max_ = 0.34 e Å^−3^
Δρ_min_ = −0.29 e Å^−3^
Absolute structure: Flack (1985 ▶), 1535 Friedel pairs
Flack parameter: −0.06 (6)

### 

Data collection: *APEX2* (Bruker, 2009 ▶); cell refinement: *SAINT-Plus* (Bruker, 2009 ▶); data reduction: *SAINT-Plus*; program(s) used to solve structure: *SHELXS97* (Sheldrick, 2008 ▶); program(s) used to refine structure: *SHELXL97* (Sheldrick, 2008 ▶); molecular graphics: *ORTEP-3 for Windows* (Farrugia, 1997 ▶); software used to prepare material for publication: *WinGX* (Farrugia, 1999 ▶).

## Supplementary Material

Crystal structure: contains datablocks I, global. DOI: 10.1107/S1600536810034070/er2079sup1.cif

Structure factors: contains datablocks I. DOI: 10.1107/S1600536810034070/er2079Isup2.hkl

Additional supplementary materials:  crystallographic information; 3D view; checkCIF report

## Figures and Tables

**Fig. 1.**
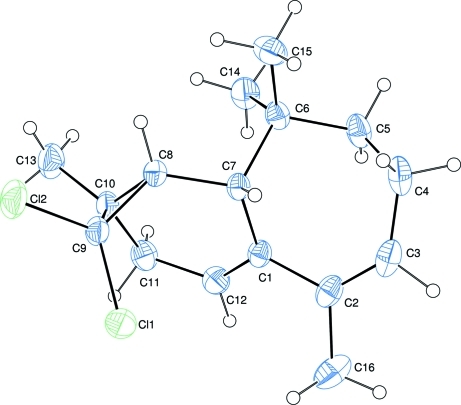
: Molecular structure of the title compound with the atom-labelling scheme. Displacement ellipsoids are drawn at the 30% probability. level. H atoms are represented as small spheres of arbitrary radii.

**Fig. 2.**
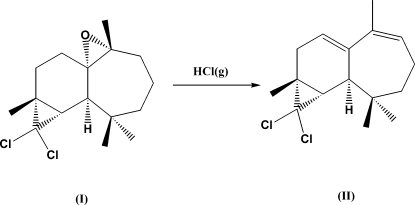
The formation of the title compound.

## supplementary crystallographic information

### Comment

Our work lies within the framework of the valorization of the most abundant essential oils in Morocco, such as Cedrus atlantica. This oil is made up mainly (75%) of bicyclic sesquiterpenes hydrocarbons, among which is found the compound, β-himachalene (Bisarya & Dev, 1968; Plattier & Teiseire, 1974). The reactivity of this sesquiterpene has been studied extensively by our team (El Jamili *et al.*, 2002; Sbai *et al.*, 2002) in order to prepare new products having olfactive proprieties suitable for the perfume or cosmetics industry. Thus, the action of one equivalent of *meta*-chloroperbenzoïc acide (*m*-CPBA) on, β- himachalène gives in quantitative yields the monoepoxyde (Benharref *et al.*, 1991; Chekroun *et al.*, 2000). The treatement of this monoepoxyde with dichlorocarbene, generated *in situ* from chloroform and in the presence of sodium hydroxide as base and n-benzyltriethylammonium chloride as catalyst, give a mixtrure of two diastereoisomers: (1*S*,2*R*,7*S*,8S,10*R*) -9,9-dichloro-1,2- epoxy-2,6,6,10-tetramethyl-tricyclo[5,5,0,08,10]dodecane and (1*S*,2*R*,7*S*,8*R*,10*S*) -9,9-dichloro-1,2-epoxy-2,6,6,10-tetramethyl- tricyclo[5,5,0,0^8,10^] dodecane (Lassaba *et al.*, 1997). Also in order to prepare products with high added value, we have treated the isomer (1*S*,2*R*,7*S*,8S,10*R*) -9,9-dichloro-1,2- epoxy-2,6,6,10-tetramethyl-tricyclo[5,5,0,0^8,10^] dodecane (I) by hydrochloric acid gas and we got one sesquiterpene dichloro-hydrocarbure (II) in yield 75%. The molecule is built up from two fused six-and seven-membered rings(Fig.1). The six-membered ring has a perfect chair conformation, with as indicated by the total puckering amplitude QT = 0.2385 (2)Å and spherical polar angle θ= 99.60 (2)° with φ -117.07 (2)°, whereas the seven-membered ring display a screw boat conformation with QT = 0.9566 (2) Å, θ = 68.84 (2)°, φ2 = -112.42 (1)° and φ3 = 142.26 (3)° (Cremer & Pople, 1975). Owing to the presence of the Cl atoms, the absolute configuration could be fully confirmed to be C7(S), C8(S) and C10(*R*) (Flack & Bernardinelli, 2000).

### Experimental

100 mg (0,33 mm l) of the isomer, (1*S*,2*R*,7*S*,8S,10*R*)-9,9-dichloro- 1,2-epoxy-2,6,6,10- tetramethyl-tricyclo[5,5,0,08,10]dodecane, dissolved in 20 ml of dichloromethane and then treated with a stream of gaseous hydrochloric acid at 0° for 5 minutes. After concentration of solvent, the residue obtained was chromatgraphed on silica gel impregnated with silver nitrate (10%) with hexane as eluent.

### Refinement

Except H3 and H12, all H atoms were fixed geometrically and treated as riding with C—H = 0.96 Å (methyl),0.97 Å (methylene), 0.98Å (methine) with *U*_iso_(H) = 1.2Ueq(methylene, methine) or *U*_iso_(H) = 1.5Ueq(methyl).

### Crystal data

**Table:**

C_16_H_22_Cl_2_	*F*(000) = 608
*M**_r_* = 285.24	*D*_x_ = 1.271 Mg m^−^^3^
Orthorhombic, *P*2_1_2_1_2_1_	Mo *K*α radiation, λ = 0.71073 Å
Hall symbol: P 2ac 2ab	Cell parameters from 10992 reflections
*a* = 7.4356 (17) Å	θ = 1.7–28.4°
*b* = 8.3124 (18) Å	µ = 0.42 mm^−^^1^
*c* = 24.108 (6) Å	*T* = 298 K
*V* = 1490.1 (6) Å^3^	Prism, colourless
*Z* = 4	0.27 × 0.18 × 0.12 mm

### Data collection

**Table:**

Bruker X8 APEXII CCD area-detector diffractometer	3282 reflections with *I* > 2σ(*I*)
Radiation source: fine-focus sealed tube	*R*_int_ = 0.026
graphite	θ_max_ = 28.4°, θ_min_ = 1.7°
φ and ω scans	*h* = −7→9
10992 measured reflections	*k* = −8→11
3691 independent reflections	*l* = −32→28

### Refinement

**Table:**

Refinement on *F*^2^	Secondary atom site location: difference Fourier map
Least-squares matrix: full	Hydrogen site location: inferred from neighbouring sites
*R*[*F*^2^ > 2σ(*F*^2^)] = 0.032	H atoms treated by a mixture of independent and constrained refinement
*wR*(*F*^2^) = 0.096	*w* = 1/[σ^2^(*F*_o_^2^) + (0.0579*P*)^2^] where *P* = (*F*_o_^2^ + 2*F*_c_^2^)/3
*S* = 1.11	(Δ/σ)_max_ = 0.001
3691 reflections	Δρ_max_ = 0.34 e Å^−^^3^
175 parameters	Δρ_min_ = −0.29 e Å^−^^3^
0 restraints	Absolute structure: Flack (1985), 1535 Friedel pairs
Primary atom site location: structure-invariant direct methods	Flack parameter: −0.06 (6)

### Special details

**Table:**

Geometry. All e.s.d.'s (except the e.s.d. in the dihedral angle between two l.s. planes) are estimated using the full covariance matrix. The cell e.s.d.'s are taken into account individually in the estimation of e.s.d.'s in distances, angles and torsion angles; correlations between e.s.d.'s in cell parameters are only used when they are defined by crystal symmetry. An approximate (isotropic) treatment of cell e.s.d.'s is used for estimating e.s.d.'s involving l.s. planes.

### Fractional atomic coordinates and isotropic or equivalent isotropic displacement parameters (Å^2^)

**Table:**

	*x*	*y*	*z*	*U*_iso_*/*U*_eq_	
C1	0.6213 (2)	0.33418 (19)	0.89982 (6)	0.0375 (3)	
C2	0.4484 (2)	0.3173 (2)	0.92944 (6)	0.0476 (4)	
C3	0.3427 (3)	0.4400 (3)	0.94246 (7)	0.0551 (5)	
C4	0.3834 (3)	0.6154 (3)	0.93795 (8)	0.0588 (5)	
H4A	0.3146	0.6730	0.9658	0.071*	
H4B	0.3451	0.6535	0.9018	0.071*	
C5	0.5809 (2)	0.6534 (2)	0.94551 (7)	0.0514 (4)	
H5A	0.6290	0.5834	0.9741	0.062*	
H5B	0.5919	0.7633	0.9587	0.062*	
C6	0.6957 (2)	0.63474 (18)	0.89326 (6)	0.0405 (3)	
C7	0.6413 (2)	0.47755 (17)	0.86197 (6)	0.0341 (3)	
H7	0.5234	0.4970	0.8451	0.041*	
C8	0.7727 (2)	0.44941 (18)	0.81513 (6)	0.0384 (3)	
H8	0.8138	0.5487	0.7973	0.046*	
C9	0.7663 (2)	0.30866 (19)	0.77693 (7)	0.0427 (3)	
C10	0.9167 (2)	0.3203 (2)	0.81854 (7)	0.0438 (3)	
C11	0.9108 (2)	0.2114 (2)	0.86840 (8)	0.0497 (4)	
H11A	0.9257	0.1014	0.8558	0.060*	
H11B	1.0127	0.2368	0.8920	0.060*	
C12	0.7456 (3)	0.2200 (2)	0.90240 (7)	0.0457 (4)	
C13	1.1028 (3)	0.3568 (3)	0.79848 (9)	0.0628 (5)	
H13A	1.1600	0.2590	0.7867	0.094*	
H13B	1.1712	0.4044	0.8280	0.094*	
H13C	1.0966	0.4304	0.7679	0.094*	
C14	0.8912 (3)	0.6291 (2)	0.91128 (8)	0.0546 (4)	
H14A	0.9119	0.5336	0.9328	0.082*	
H14B	0.9181	0.7221	0.9334	0.082*	
H14C	0.9673	0.6280	0.8791	0.082*	
C15	0.6651 (3)	0.7795 (2)	0.85566 (8)	0.0573 (5)	
H15A	0.7378	0.7690	0.8229	0.086*	
H15B	0.6978	0.8761	0.8750	0.086*	
H15C	0.5406	0.7847	0.8453	0.086*	
C16	0.3883 (3)	0.1489 (3)	0.94230 (11)	0.0727 (6)	
H16A	0.4725	0.0996	0.9673	0.109*	
H16B	0.3825	0.0876	0.9086	0.109*	
H16C	0.2716	0.1518	0.9593	0.109*	
Cl1	0.59995 (6)	0.16140 (5)	0.781164 (18)	0.05350 (13)	
Cl2	0.81596 (8)	0.34714 (7)	0.706600 (17)	0.06577 (16)	
H3	0.223 (3)	0.414 (3)	0.9587 (9)	0.074 (6)*	
H12	0.732 (3)	0.127 (3)	0.9270 (9)	0.064 (6)*	

### Atomic displacement parameters (Å^2^)

**Table:**

	*U*^11^	*U*^22^	*U*^33^	*U*^12^	*U*^13^	*U*^23^
C1	0.0388 (7)	0.0457 (7)	0.0281 (6)	−0.0004 (7)	0.0008 (5)	−0.0004 (6)
C2	0.0430 (9)	0.0676 (11)	0.0323 (7)	−0.0041 (8)	0.0017 (6)	0.0061 (7)
C3	0.0393 (9)	0.0868 (13)	0.0391 (9)	−0.0009 (9)	0.0065 (7)	−0.0031 (9)
C4	0.0457 (10)	0.0781 (12)	0.0526 (10)	0.0142 (9)	0.0084 (8)	−0.0187 (9)
C5	0.0500 (9)	0.0632 (10)	0.0411 (8)	0.0055 (9)	−0.0002 (7)	−0.0196 (8)
C6	0.0404 (8)	0.0440 (8)	0.0370 (7)	0.0023 (7)	−0.0012 (6)	−0.0080 (6)
C7	0.0324 (7)	0.0403 (7)	0.0297 (6)	0.0027 (6)	−0.0007 (5)	−0.0020 (5)
C8	0.0422 (8)	0.0404 (7)	0.0326 (7)	−0.0017 (6)	0.0054 (6)	−0.0022 (6)
C9	0.0434 (8)	0.0512 (8)	0.0334 (7)	−0.0048 (6)	0.0071 (6)	−0.0074 (6)
C10	0.0374 (8)	0.0520 (8)	0.0420 (8)	0.0002 (7)	0.0066 (6)	−0.0116 (7)
C11	0.0441 (9)	0.0517 (9)	0.0532 (10)	0.0111 (7)	−0.0005 (8)	−0.0033 (7)
C12	0.0526 (10)	0.0458 (8)	0.0388 (8)	0.0016 (7)	−0.0013 (7)	0.0033 (7)
C13	0.0432 (9)	0.0831 (13)	0.0621 (11)	−0.0044 (10)	0.0113 (8)	−0.0171 (11)
C14	0.0431 (9)	0.0663 (11)	0.0545 (10)	−0.0054 (9)	−0.0050 (8)	−0.0144 (8)
C15	0.0744 (14)	0.0392 (8)	0.0583 (11)	0.0027 (9)	−0.0033 (10)	−0.0024 (8)
C16	0.0590 (12)	0.0815 (13)	0.0776 (14)	−0.0113 (12)	0.0084 (11)	0.0310 (12)
Cl1	0.0529 (2)	0.0574 (2)	0.0502 (2)	−0.0120 (2)	−0.00032 (18)	−0.00975 (19)
Cl2	0.0750 (3)	0.0874 (3)	0.0349 (2)	−0.0115 (3)	0.0142 (2)	−0.0107 (2)

### Geometric parameters (Å, °)

**Table:**

C1—C12	1.326 (2)	C9—C10	1.505 (2)
C1—C2	1.477 (2)	C9—Cl1	1.7433 (16)
C1—C7	1.508 (2)	C9—Cl2	1.7645 (17)
C2—C3	1.326 (3)	C10—C13	1.497 (2)
C2—C16	1.502 (3)	C10—C11	1.505 (3)
C3—C4	1.493 (3)	C11—C12	1.479 (3)
C3—H3	1.00 (3)	C11—H11A	0.9700
C4—C5	1.513 (3)	C11—H11B	0.9700
C4—H4A	0.9700	C12—H12	0.98 (2)
C4—H4B	0.9700	C13—H13A	0.9600
C5—C6	1.530 (2)	C13—H13B	0.9600
C5—H5A	0.9700	C13—H13C	0.9600
C5—H5B	0.9700	C14—H14A	0.9600
C6—C14	1.519 (2)	C14—H14B	0.9600
C6—C15	1.524 (2)	C14—H14C	0.9600
C6—C7	1.562 (2)	C15—H15A	0.9600
C7—C8	1.511 (2)	C15—H15B	0.9600
C7—H7	0.9800	C15—H15C	0.9600
C8—C9	1.490 (2)	C16—H16A	0.9600
C8—C10	1.518 (2)	C16—H16B	0.9600
C8—H8	0.9800	C16—H16C	0.9600
			
C12—C1—C2	121.04 (16)	C8—C9—Cl2	116.42 (12)
C12—C1—C7	121.66 (14)	C10—C9—Cl2	118.25 (11)
C2—C1—C7	116.97 (14)	Cl1—C9—Cl2	109.39 (9)
C3—C2—C1	123.86 (17)	C13—C10—C11	114.00 (16)
C3—C2—C16	119.48 (18)	C13—C10—C9	118.97 (15)
C1—C2—C16	116.59 (17)	C11—C10—C9	118.17 (15)
C2—C3—C4	127.88 (17)	C13—C10—C8	119.41 (16)
C2—C3—H3	117.2 (14)	C11—C10—C8	116.60 (13)
C4—C3—H3	114.8 (13)	C9—C10—C8	59.03 (10)
C3—C4—C5	113.09 (17)	C12—C11—C10	115.96 (15)
C3—C4—H4A	109.0	C12—C11—H11A	108.3
C5—C4—H4A	109.0	C10—C11—H11A	108.3
C3—C4—H4B	109.0	C12—C11—H11B	108.3
C5—C4—H4B	109.0	C10—C11—H11B	108.3
H4A—C4—H4B	107.8	H11A—C11—H11B	107.4
C4—C5—C6	114.91 (14)	C1—C12—C11	126.00 (17)
C4—C5—H5A	108.5	C1—C12—H12	121.5 (13)
C6—C5—H5A	108.5	C11—C12—H12	112.4 (13)
C4—C5—H5B	108.5	C10—C13—H13A	109.5
C6—C5—H5B	108.5	C10—C13—H13B	109.5
H5A—C5—H5B	107.5	H13A—C13—H13B	109.5
C14—C6—C15	109.72 (16)	C10—C13—H13C	109.5
C14—C6—C5	107.57 (14)	H13A—C13—H13C	109.5
C15—C6—C5	109.07 (14)	H13B—C13—H13C	109.5
C14—C6—C7	111.10 (13)	C6—C14—H14A	109.5
C15—C6—C7	109.56 (13)	C6—C14—H14B	109.5
C5—C6—C7	109.78 (13)	H14A—C14—H14B	109.5
C1—C7—C8	113.17 (12)	C6—C14—H14C	109.5
C1—C7—C6	113.22 (12)	H14A—C14—H14C	109.5
C8—C7—C6	108.85 (12)	H14B—C14—H14C	109.5
C1—C7—H7	107.1	C6—C15—H15A	109.5
C8—C7—H7	107.1	C6—C15—H15B	109.5
C6—C7—H7	107.1	H15A—C15—H15B	109.5
C9—C8—C7	124.25 (13)	C6—C15—H15C	109.5
C9—C8—C10	60.05 (10)	H15A—C15—H15C	109.5
C7—C8—C10	121.66 (13)	H15B—C15—H15C	109.5
C9—C8—H8	113.6	C2—C16—H16A	109.5
C7—C8—H8	113.6	C2—C16—H16B	109.5
C10—C8—H8	113.6	H16A—C16—H16B	109.5
C8—C9—C10	60.92 (11)	C2—C16—H16C	109.5
C8—C9—Cl1	122.54 (11)	H16A—C16—H16C	109.5
C10—C9—Cl1	122.22 (12)	H16B—C16—H16C	109.5

## References

[bb1] Benharref, A., Chekroun, A. & Lavergne, J. P. (1991). *Bull. Soc. Chim. Fr.* **128**, 738–741.

[bb2] Bisarya, S. C. & Dev, S. (1968). *Tetrahedron*, **24**, 3861–3867.

[bb3] Bruker (2009). *APEX2* and *SAINT-Plus* Bruker AXS Inc., Madison, Wisconsin, USA.

[bb4] Chekroun, A., Jarid, A., Benharref, A. & Boutalib, A. (2000). *J. Org. Chem.* **65**, 4431–4434. 10.1021/jo991848c 10891148

[bb5] Cremer, D. & Pople, J. A. (1975). *J. Am. Chem. Soc.* **97**, 1354–1358.

[bb6] El Jamili, H., Auhmani, A., Dakir, M., Lassaba, E., Benharref, A., Pierrot, M., Chiaroni, A. & Riche, C. (2002). *Tetrahedron Lett.* **43**, 6645–6648.

[bb7] Farrugia, L. J. (1997). *J. Appl. Cryst.* **30**, 565.

[bb8] Farrugia, L. J. (1999). *J. Appl. Cryst.* **32**, 837–838.

[bb9] Flack, H. D. (1983). *Acta Cryst.* A**39**, 876–881.

[bb10] Lassaba, E., Chekroun, A., Benharref, A., Chiaroni, A., Riche, C. & Lavergne, J.-P. (1997). *Bull. Soc. Chim. Belg.* **106**, 281–288.

[bb11] Plattier, M. & Teiseire, P. (1974). *Recherche*, **19**, 131–144.

[bb12] Sbai, F., Dakir, M., Auhmani, A., El Jamili, H., Akssira, M., Benharref, A., Kenz, A. & Pierrot, M. (2002). *Acta Cryst.* C**58**, o518–o520. 10.1107/s0108270102012283 12154317

[bb13] Sheldrick, G. M. (2008). *Acta Cryst.* A**64**, 112–122. 10.1107/S0108767307043930 18156677

